# A Single Postnatal Dose of Dexamethasone Enhances Memory of Rat Pups Later in Life

**DOI:** 10.1371/journal.pone.0165752

**Published:** 2016-10-31

**Authors:** Kuen-Jer Tsai, Chun-I Sze, Yung-Chieh Lin, Yuh-Jyh Lin, Ting-Hui Hsieh, Chyi-Her Lin

**Affiliations:** 1 Institute of Clinical Medicine, College of Medicine, National Cheng Kung University, Tainan, Taiwan; 2 Center of Clinical Medicine, National Cheng Kung University Hospital, College of Medicine, National Cheng Kung University, Tainan, Taiwan; 3 Department of Pathology and Cell Biology and Anatomy, College of Medicine, National Cheng Kung University, Tainan, Taiwan; 4 Department of Pediatrics, College of Medicine, National Cheng Kung University, Tainan, Taiwan; 5 Department of Pediatrics, National Cheng Kung University Hospital, College of Medicine, National Cheng Kung University, Tainan, Taiwan; Radboud University Medical Centre, NETHERLANDS

## Abstract

Postnatal dexamethasone (Dex) therapy is associated with adverse neurodevelopmental outcomes, which might be related to its timing of administration. We used time-dated pregnant Wistar *albino* rats, whose litters were divided into experimental (Dex) and control groups intraperitoneally administered one dose of Dex (0.5 mg/kg) or normal saline (NS), respectively, at either day 1 (P1) or 7 (P7). The magnitude of the contextual freezing response and performance on the Morris water maze were significantly higher in the Dex-P7 group than in those of the other groups at P56. Dendritic spine density, membranous expression of the *N*-methyl-d-aspartate receptor (NMDAR) subunit NR2A/2B, and postsynaptic density-95 (PSD-95) were significantly higher in the Dex-P7 group than in the other groups. Furthermore, cytosolic expression of nuclear factor kappa B (NF-κB) and phosphatidylinositol 3-kinase (PI3K) was significantly higher in the Dex group than in NS group. Moreover, Dex administration at P7 increased cell proliferation, neuronal differentiation, and the survival of newly born neurons in the dentate gyrus. These results suggest Dex at P7 enhances the acquisition of contextual fear and spatial memory later in life due to the modulation of the newly born neurons, increase in dendritic spine number, and NMDAR expression.

## Introduction

Dexamethasone (Dex), a synthetic glucocorticoid, has been used in preterm infants to prevent or treat bronchopulmonary dysplasia, or chronic lung disease (CLD). However, early postnatal Dex treatment has been shown to adversely affect long-term neurodevelopment in patients [1–3]. The dosage of Dex used in the moderate-early trials for reducing CLD ranged from 0.2 mg/kg to 0.5 mg/kg per day, and its duration lasted from 3 to 42 days in those studies [4]. So far, the optimal doses of Dex for preventing or treating CLD in high risk infants is undetermined [5].

Preterm infants may present with intractable hypotension, which sometimes requires a single dose of Dex for treatment [6,7]. Whether this regimen could also result in adverse neurological effects is unknown. Dex administration reduces cerebral gray matter volume, indicating that it may potentially affect certain types of neuronal cells [8–10]. The affinity of Dex for the glucocorticoid receptors (GRs) is higher than mineralocorticoid receptors (MRs) [11,12]. In addition, the effects of glucocorticoids on learning and memory may be mediated through GRs [13]. The hippocampus contains a high density of GRs, and administration of Dex early in life has resulted in changes to hippocampal synaptic plasticity and disrupts memory formation [14,15]. However, studies have also shown neuroprotective effects of Dex which are related to GRs and phosphatidylinositol 3-kinase (PI3K) [16], which is mediated by the regulatory p85 subunit of PI3K [17].

*N*-methyl-D-aspartate receptor (NMDAR) is a type of glutamate receptor. When synaptic NMDAR is activated, it can recruit more postsynaptic density-95 (PSD-95) into synapses through the PI3K pathway [18]. PSD-95 is an important postsynaptic component that regulates spine density, and the gene for PSD-95 is a critical target of nuclear factor kappa B (NF-κB) [19,20]. The p65 heterodimer of NF-κB is involved in learning and memory processes [21,22] and is required for up-regulation of dendritic spine density and synaptogenesis [19].

The brain of a P1 rat pup corresponds to a human fetus at about the 22^nd^ to the 24^th^ week of gestation, and a P7 rat pup is approximately equivalent to a full-term infant in brain growth, neurochemical data, electroencephalographic patterns, and synapse formation [23]. The rat model is useful in following up on the adverse effects of Dex, due to the relatively shorter life span of the animals, and their vulnerability to the effects of steroids. During early postnatal development, neonatal pups undergo a reduced hypothalamic-pituitary-adrenal (HPA) response to environmental stressors [24,25]. Rat pups from P4 to P14 undergo what is characterized as the stress hyporesponsive period, and glucocorticoid levels are lowest at P9 [26]. Therefore, exogenous glucocorticoids may affect glucocorticoid-sensitive tissues during this period [27,28]. Glucocorticoid receptors are enriched in the membranes of neural stem cells, whose proliferation increases after birth and reaches a maximum at P6-P8 [29].

The p65 subunit of NF-κB and PSD-95 are involved in regulating the growth of spine density during neuronal development [19,20,30], during which synaptogenesis reaches a plateau in mature cultures [19,31]. We reported that a single dose of Dex administered in P1 rat pups increases apoptosis in the dentate gyrus [12]. In contrast, this phenomenon is not prominent in P7 pups that received the same treatment. Furthermore, double immunofluorescence staining of the tissue showed that most of the apoptotic cells were neuroprogenital cells. The results indicate that timing of Dex treatment is critical for glucocorticoid induced adverse neurodevelopmental effects. A similar study that used a rat model of the disease, showed that Dex therapy changed the synaptic plasticity and disrupted memory retention in early stages, but the difference resolved by 8-weeks [14]. As synaptogenesis is still occurring in the hippocampus at P7, we hypothesize that a single dose of Dex given to P1 and P7 rat pups may induce different histological and biochemical changes of the hippocampus later in life.

## Materials and Methods

### Animals and treatments

All of the experimental protocols were approved by National Cheng Kung University Animal Ethics Committee. The care and handling of the animals were conducted in accordance with the National Institute of Health guidelines for ethical animal treatment. Time-dated pregnant Wistar *albino* rats were used in this study, we used seven dams, and picked up male pups from each dam. The male pups of each litter were randomly divided into four groups: the pups in the experimental groups (Dex) received one dose of Dex (0.5 mg/kg; Oradexon, 4 mg/mL, Organon, Netherlands) and control littermates received an equivalent volume of normal saline (NS) intraperitoneally at P1 or P7. Previous study on rats at our institution found that early changes of synaptic function induced by Dex treatment recovered at P56 [32]. Therefore, we conducted behavioral test at P56. Either acquisition in the fear-conditioning task or performance in the Morris water maze was measured at P56, and the young-adult rats were sacrificed immediately afterwards. The hippocampus was isolated from the rat’s brain and assigned to three portions. One was processed for Golgi staining, another was homogenized for Western blotting, and the third was prepared for immunofluorescence staining. The experiments were conducted according to the National Cheng Kung University Animal Ethics Committee ethical guidelines. Care and handling of the animals were in accordance with the National Institute of Health guidelines for ethical treatment of animals. All surgery was performed under anesthesia, and all efforts were made to minimize suffering of the rats.

### Fear-conditioning task

The task was performed as described previously [33]. Rats were trained and tested in a fear-conditioning chamber (Med Associates, St. Albans VT). The set-up was enclosed in a ventilated, sound-attenuating cabinet (length 38 cm, width 38 cm, and height 55 cm). The conditioned stimulus (CS) [5] was an acoustic startle stimulus, which was a 15 millisecond noise (a 95 dB sound at 1000 Hz). The unconditioned stimulus (US) was a 0.8 mA foot shock with duration of 1 s. At the acclimation phase, rats were placed in the startle test boxes for 10 min without a CS or US, and returned to their home cages for three consecutive days. At the training phase, rats were placed in the startle boxes and received 10 sound/foot shock pairings with an inter-trial interval (ITI) of 2 min. Twenty-four hours after training, rats were tested for a freezing response. The contextual fear-conditioning task was measured in different boxes without a CS or US, and the cued fear-conditioning task was measured in another box, in which only the CS was presented. The test was presented in a balanced mixed order. The cut-off time was 180 s per trial and the interval time between each trial was 30 s. The video taken during the test was analyzed by FreezeScan (Clever Sys Inc.) and the percentage of freezing score was calculated as: (freezing time/total observed time) ×100% to normalize the fold of the freezing time over the total observed time by percentage values.

### Morris Water Maze

The task was performed as described previously [34]. Briefly, animals were trained to learn the location of a hidden platform in a pool of water (180 cm diameter, 50 cm depth). The escape platform (10 cm diameter) was submerged 4 cm below the water surface. Visual cues were located on the wall of the pool. The animals were subjected to four trials per session and a total of 6 sessions. The time spent by the individual animals to reach the platform in the water, was recorded as the escape latency. The cut-off time was 120 s per trial and the interval time between each trial was 20 s. After training, the platform was removed and the rats were immediately examined using a probe test. The proportion of time spent searching in the target quadrant was measured, and the number of times the platform location was crossed was also counted. To check for inter-observer reliability, the video for the probe test was blindly scored and analyzed by two observers. The inter-observer reliability was highly significant (r = 0.98) and the remainder of the trials were analyzed by one of the two observers. To check for intra-observer reliability, the same observer re-scored the video and the intra-observer reliability was higher than 95% (r = 0.99).

### Golgi staining

The hippocampi were processed for Golgi-Cox staining to visualize dendritic spine structure in neurons. Rats were perfused transcardially with 0.01 M phosphate buffered saline (PBS, pH 7.4), and extracted brain tissues were stored in Golgi-Cox solution for 14 days in the dark at room temperature (renew Golgi-cox solution at the second day) and then transferred to 30% sucrose solution. Coronal sections were cut at a 200-μm thickness and stained by immersing them in NH_4_OH followed by Kodak fixative solution, each for 30 min. The sections were dehydrated in gradient ethanol, cleared in xylene, and then coverslipped. Sections were examined by light microscopy and the images captured by a video camera (Eclipse 80i, Nikon, Japan) coupled to a desktop computer. Number of spines, dendrite length, and branching of observed dendrites were quantified and measured by Image Pro-Plus 4.5 software (Media Cybernetics, Silver Spring, USA). The quantification was carried out by an experimenter blinded to the experimental condition. Spine density was computed as (number of spines in observed dendrites/length of observed dendrites). Each image was quantified by two observers to check for inter-observer reliability, which was r = 0.97. The same observer re-scored the images for each task and the intra-observer reliability was r = 0.97.

### Western blotting

The hippocampi were dissected and protein homogenates were prepared from the rats at P56, immediately after the acquisition of contextual and cued fear-conditioning tasks were completed. Western blot analysis was performed on membranous and cytosolic fractions of the hippocampi homogenates as described previously [35]. Briefly, 20 μg protein homogenate, determined by the Bradford protein assay (Bio-Rad, Hercules, CA), was separated by SDS-PAGE, blotted onto nitrocellulose (Hy-Bond, Amersham, Arlington Heights, IL), and blocked with nonfat dry milk. Blots were incubated with a specific primary antibody. The following primary antibodies were used: mouse monoclonal GluR1 (1:1000; Santa Cruz Biotechnology, Santa Cruz, CA; SC-13152) and GluR2 (1:1000; Santa Cruz Biotechnology; SC-7610), goat polyclonal NR2A (1:2000; Santa Cruz Biotechnology; SC-1468), rabbit NR2B (1:5000; Cell Signaling Technology; No. D15B3), polyclonal rabbit PSD-95 (1:50000; Abcam, Cambridge, UK; ab18258), rabbit PI3Kp110 and PI3Kp85 (1:1000; Cell Signaling Technology; No. 4255s and 4292s, respectively), and rabbit NF-κBp65 (1:1000; Santa Cruz Biotechnology; SC-372). The blots were followed by incubation with horseradish-peroxidase-conjugated secondary antibodies (Perkin Elmer, Waltham, MA) and detection using enhanced chemiluminescence (ECL; Bio-Rad). Each experiment was performed at least three times. The intensities of specific bands were measured with ImageJ (NIH, USA). The relative intensities of the bands were normalized against that of tubulin and expressed as means ± standard error of the mean (SEM). Each optic intensity was quantified by two observers and the inter-observer reliability was r = 0.99. The same observer re-quantified the optic intensity and the intra-observer reliability was r = 0.98.

### BrdU labeling

BrdU (Roche, Indianapolis, Indiana, USA) is a thymidine analogue that is incorporated into the DNA of dividing cells during mitosis. To label the newly born cells, P7 rat pups were administrated a pulse of BrdU (50 mg/kg body weight) intraperitoneally (i.p.) after either NS or Dex.

### Immunofluorescence staining

Rats were anesthetized and euthanized with 10% chloral hydrate (400 mg/kg, i.p.) and then perfused transcardially with 0.01 M PBS, followed by 4% paraformaldehyde in 0.01 M PBS (pH 7.4). The brains were postfixes in 4% paraformaldehyde for 48 h and embedded in paraffin. The tissue blocks were cut into 7 μm-thick coronal sections, and sections were deparaffinized and rehydrated before immunofluorescence staining. For detecting BrdU, sections were incubated with 2N HCl at 37°C for 15 min and neutralized with 0.1M boric acid (pH 8.5; Sigma, St. Louis, MO, USA). The sections were blocked with 5% normal donkey serum (Millipore, Temecula, CA) and 0.1% Triton X-100 (Sigma) in PBS. The monoclonal anti-BrdU (1:200; Abcam; ab6326) was used to label newly born nuclei. Alexa Fluor 488-conjugated anti-rat antibody (Invitrogen Life Technologies, Grand Island, NY) was used to visualize the signal. To identify neurogenesis in the dentate gyrus, double immunofluorescence staining was performed to detect anti-BrdU (1:200; Abcam) and anti-NeuN (1:200; Millipore; MAB377). The monoclonal anti-NeuN was used to label the nuclei of mature neurons. Alexa Fluor 488-conjugated anti-rat antibody (Invitrogen Life Technologies) and Alexa Fluor 555-conjugated anti-mouse antibody (Invitrogen Life Technologies) were used respectively. Sections were examined by fluorescent microscopy (BX51, Olympus, Japan) and the images captured by a camera coupled to a desktop computer. Quantification was performed by a viewer blinded to the experimental group. The number of cells was counted using TissueQuest software (Tissue Gnostics, Vienna, Austria).

### Statistical Analysis

Quantitative results are expressed as mean ± SEM. Statistical analyses were performed using a two-tail nonparametric Mann-Whitney test, or a two-way analysis of variance [36] with a Bonferroni multiple comparisons test. A *p* value < 0.05 was considered statistically significant.

## Results

### Improved performance of young-adult rats in fear-conditioning tasks and Morris water maze following Dex administration at postnatal day 7

**Fig 1:**
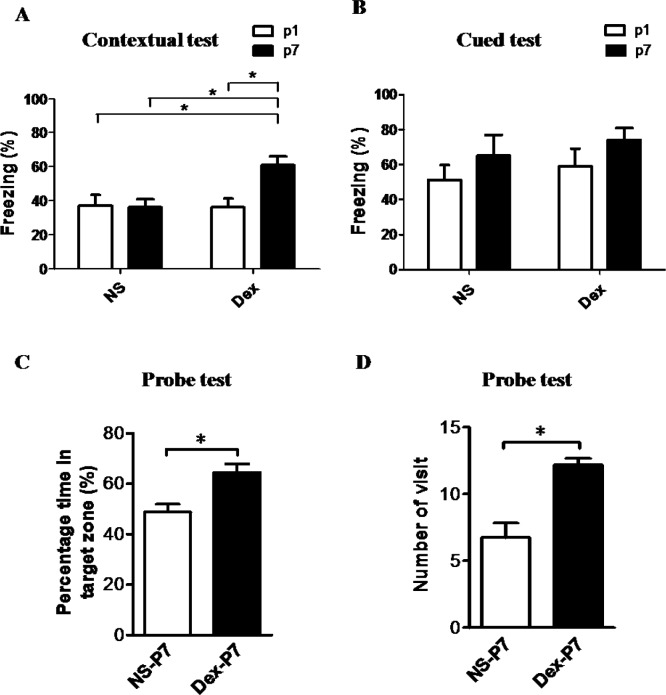
Effects of postnatal administration of dexamethasone (Dex) on fear memory and spatial learning memory formation. Dex was administered at P1 or P7 and behavioral tasks were performed at P56. (A) The freezing response in contextual fear-conditioning task was significantly greater in the Dex-P7 group than in the other groups (two-way ANOVA with Bonferroni multiple comparisons test, *n* = 8 for each group *p < 0.05), and Dex-P7 group was significantly greater than NS-P7 group (F[1,28] = 4.59, *p < 0.05). (B) The freezing response in the cued fear-conditioning task was not significantly altered among the four groups (*n* = 8 for each group). (C) The proportion of time spent searching in target quadrant was significantly higher in the Dex group (64.50 ± 1.50) than in the NS group (48.75 ± 1.56) (Mann-Whitney test, *n* = 5 for each group, *p < 0.05). (D) The number of crossings in the platform location was higher in the Dex group (12.20 ± 0.22) than in the NS group (6.75 ± 0.55) (Mann-Whitney test, *n* = 5 for each group, *p < 0.05). The data are expressed as mean ± SEM of three independent experiments.

**Fig 2:**
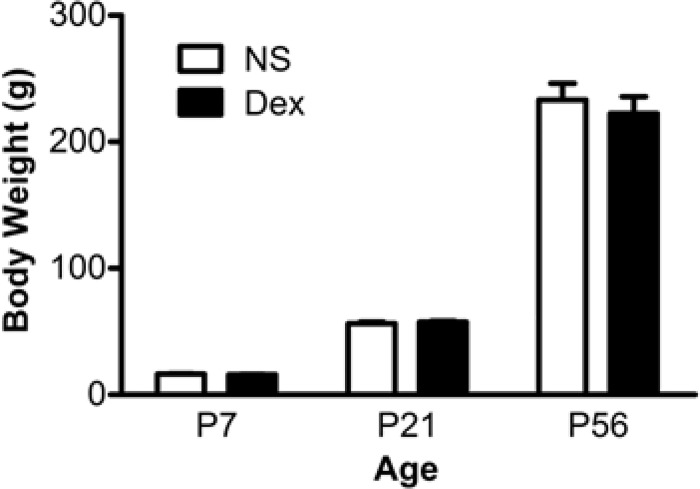
Average body weight at different ages. The pups received either normal saline (NS) or dexamethasone (Dex) at P7. Body weight was recorded before drug administration (P7), at weaning (P21), and at the time of the behavioral tests (P56). The rats continued to gain body weight with age, and there was no significant difference between NS and Dex groups. The data are expressed as mean ± SEM of three independent experiments (*n* = 5 for each group).

Rats were assessed on the contextual fear-conditioning task (hippocampus-dependent) and the cued fear-conditioning task (hippocampus-independent) (Fig 1). We recorded the freezing response of NS or Dex rats at 8 weeks of age (P56). The percentage of contextual freezing response in the Dex-P7 group was significantly higher than in the other groups (Fig 1A), *n =* 8, Dex-P1 group vs Dex-P7 group, F[1,28] = 4.60, *p < 0.05, and Dex-P7 group vs NS-P7 group, F[1,28] = 4.59, *p < 0.05. In contrast, the cued freezing response showed no significant statistical difference among the four groups (Fig 1B), Dex-P1 group vs Dex-P7 group, F[1,28] = 2.31, *p* = 0.140, NS-P7 group vs Dex-P7 group, F[1,28] = 0.78, *p* = 0.385. To confirm the effect of Dex on the function of the hippocampus, we tested performance in the Morris water maze, a more specific measurement of spatial memory. As expected, the proportion of time spent searching in the target quadrant and the number of times the platform location was crossed, were significantly higher in the Dex-P7 group than in the NS-P7 group (Fig 1C and 1D, *n =* 5, *p < 0.05). We also recorded the body weight of NS-P7 and Dex-P7 rats to exclude side effects of Dex. There was no significant difference in body weight at P7, P21 or P56 (Fig 2, *n =* 5).

### Dex increases dendritic spine density in granular cells

**Fig 3:**
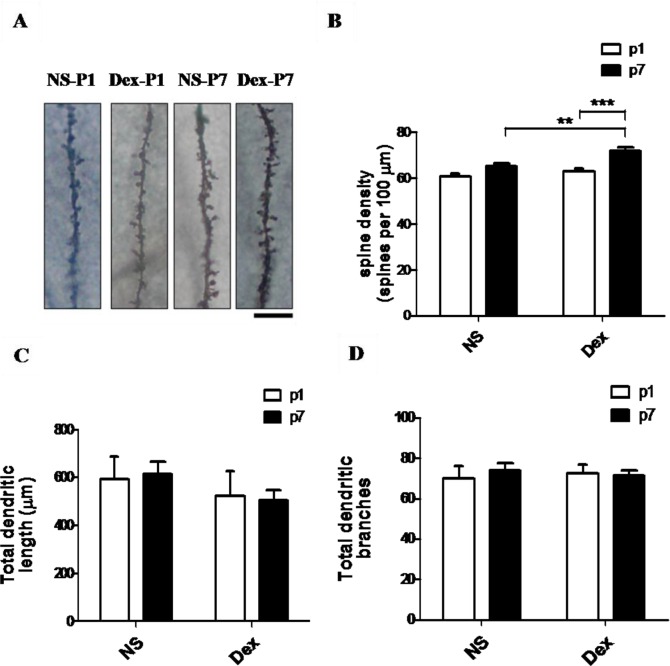
The effect of dexamethasone (Dex) on synaptic plasticity in the hippocampus. The pups received either normal saline (NS) or Dex at P1 or P7, and were sacrificed at P56 after the behavioral tests. (A) The brain sections were Golgi-stained and photomicrographs show the dendrites of granular cells. (B) The dendritic spine density of granular cells was significantly higher in the Dex-P7 group than in the other groups (two-way ANOVA with Bonferroni multiple comparisons test, ***p < 0.001). (C, D) The total dendritic length (C) and total branch numbers of the dendrites (D) did not differ significantly among the four groups. The data are expressed as mean ± SEM of three independent experiments (*n* = 6 for each group). Bar = 10 μm.

The increased response in the contextual fear-conditioning task suggested an association with dendritic regulation. We therefore investigated whether Dex administration affected dendritic spine growth in the hippocampus of rat pups. (Fig 3A) shows Golgi-stained granular cells in 8-week-old rats after administering NS or Dex at P1 or P7. The dendritic spine density of granular cells was significantly higher in rats from the Dex-P7 group than in those from the other groups (Fig 3B), *n =* 6, Dex-P7 group vs Dex-P1 group, F[1,28] = 9.05, **p < 0.01, and Dex-P7 group vs NS-P7 group, F[1,28] = 21.16, *p < 0.01. However, the total dendritic length (Dex-P7 group vs NS-P7 group, F[1,20] = 1.34, *p* = 0.260; Dex-P7 group vs Dex-P1 group, F[1,20] = 0.00, *p* = 0.995) and total number of dendritic branches (Dex-P7 group vs NS-P7 group, F[1,20] = 0.00, *p* = 0.969; Dex-P7 group vs Dex-P1 group, F[1,20] = 0.12, *p* = 0.728) did not differ among the four groups (Fig 3C and 3D).

### The expression of PSD-95 is positively correlated with dendritic spine density

**Fig 4:**
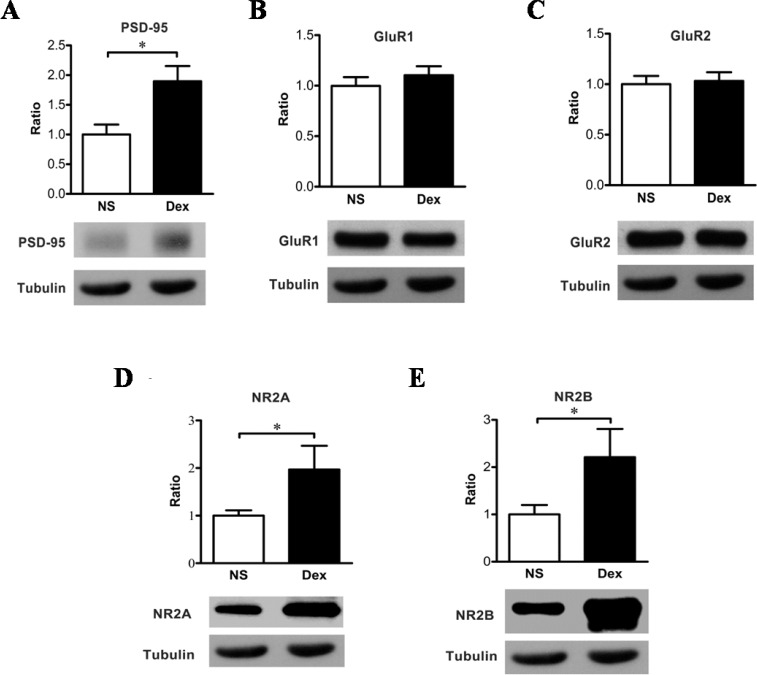
(A) Western blot analysis shows the membranous levels of PSD-95 in the hippocampus of young-adult rats receiving dexamethasone (Dex) at P7. PSD-95 levels were significantly higher in the Dex group (1.90 ± 0.11) than in the NS group (1.00 ± 0.07) (Mann-Whitney test, *p < 0.05). Tubulin was used as an internal control. The data are expressed as mean ± SEM of three independent experiments (*n* = 6 for each group). Administration of dexamethasone (Dex) at P7 does not affect the membranous level of AMPA receptors in the hippocampus measured at P56. (B-E) The membranous level of AMPA receptor subunits, GluR1 (B) and GluR2 (C), did not differ significantly between the Dex and NS groups. The expression levels of NR2A (D) and NR2B (E) were significantly higher in the Dex group (NR2A: 1.97 ± 0.20; NR2B: 2.21 ± 0.24) than in the NS group (NR2A: 1.00 ± 0.05; NR2B: 1.00 ± 0.08) (Mann-Whitney test, *p < 0.05). Tubulin was used as an internal control. These measurements were done after the contextual or cue-feared conditioning. The data are expressed as mean ± SEM of three independent experiments (*n =* 6 each group).

PSD-95, a specialized scaffold protein, is associated with the postsynaptic spine density, and is characterized as an important regulator of synaptic structure and function. After Dex administration at P7, the expression level of PSD-95 was significantly higher in the Dex group than in the control group (Fig 4A, *n =* 6, *p < 0.05), indicating that there may be a correlation between PSD-95 and dendritic spine density.

### Dex increases the surface expression of NMDA receptors in the hippocampus

Subsequently, we investigated whether the increased response in a contextual fear-conditioning task after Dex treatment at P7 was influenced by the regulation of glutamate receptors. Using western blot analysis, membrane fractions of the rat hippocampus were used to quantify GluR1 and GluR2, two subunits of the α-amino-3-hydroxy-5-methyl-4-isoxazolepropionic acid (AMPA) receptor (Fig 4B and 4C). Neither GluR1 (Fig 4B) nor GluR2 (Fig 4C) differed between the Dex and control groups, suggesting that the increased response of contextual fear-conditioning task in the Dex group was not associated with AMPA receptors directly. We then quantified NMDA receptors (NR2A and NR2B) again using western blot analysis (Fig 4D and 4E). The expression of NMDA receptors was greater in the Dex group than in the control group (Fig 4D and 4E, *p < 0.05). This data suggests that NMDA, but not AMPA receptors, may be responsible for the increased response in the contextual fear-conditioning task.

### Dex regulates PI3K and NF-κB cascades

**Fig 5:**
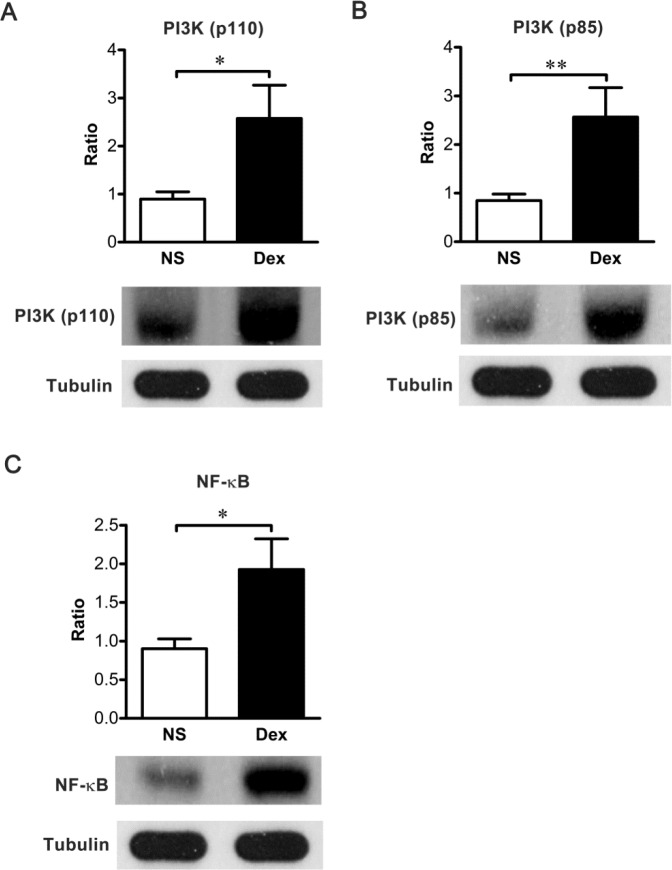
The effects of dexamethasone (Dex) are mediated through the PI3K and NF-κB pathway. Rat pups were intraperitoneally injected with Dex or normal saline (NS) at P7 and sacrificed at P56 immediately after behavioral tests. The cytosolic levels of PI3Kp110 (A), PI3Kp85 (B), and NF-κB (C) were significantly higher in the Dex group (PI3Kp110: 2.57 ± 0.22; PI3Kp85: 2.57 ± 0.19; NF-κB: 1.93 ± 0.13) than in the NS group (PI3Kp110: 0.90 ± 0.05; PI3Kp85: 0.85 ± 0.04; NF-κB: 0.90 ± 0.04) (Mann-Whitney test, *p < 0.05, **p < 0.01). The band intensities were normalized to tubulin. Results represent the mean ± SEM of three independent experiments (*n =* 10 for each group).

To understand the mechanisms underlying the effects of Dex treated at P7, we evaluated the alterations of PI3K and NF-κB in the rat hippocampus by western blotting. Both the p110 catalytic subunit and the p85 regulatory subunit of PI3K in the hippocampal lysate, were significantly increased in the Dex group (Fig 5A and 5B, *n =* 10 *p < 0.05; **p < 0.01). In addition, the level of NF-κB p65 was significantly higher in the Dex group than in the NS group (Fig 5C, *n =* 10 *p < 0.05). This data suggests that Dex acts through PI3K and NF-κB mechanisms.

### Dex increases cell proliferation in the dentate gyrus

**Fig 6:**
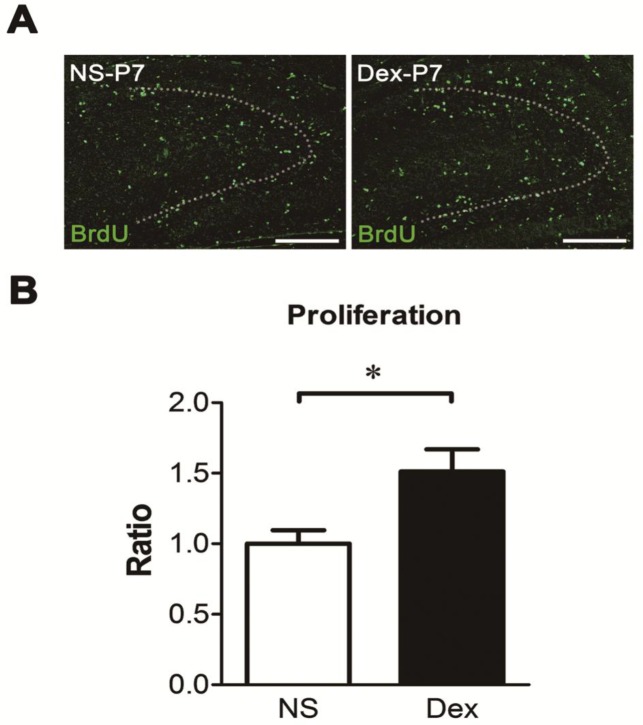
The effect of dexamethasone (Dex) on cell proliferation of dentate gyrus at P7. The rat pups were intraperitoneally injected with either NS or Dex, followed by BrdU at P7 and sacrificed 24 h later. Representative images of BrdU-labeled cells (green color) are shown in (A). The dotted lines show the shape of dentate gyrus. Bar = 200 μm. (B) Quantification of BrdU-positive nuclei indicated greater cell proliferation in the Dex-treated group than in the NS group (Mann-Whitney test, *p < 0.05). Results are representative of the mean ± SEM (*n =* 5 for each group).

To determine whether Dex treatment at P7 affects cellular proliferation in the dentate gyrus, the newly born nuclei were labeled with BrdU at P7, and the pups were sacrificed 24 h later (Fig 6A). Cell proliferation in the dentate gyrus was significantly higher in the Dex group than in the NS group (Fig 6B, *p < 0.05). This suggests that Dex produces its effects mainly in the cellular population undergoing proliferation at P7.

### Dex increases cell survival and neurogenesis in the dentate gyrus

**Fig 7:**
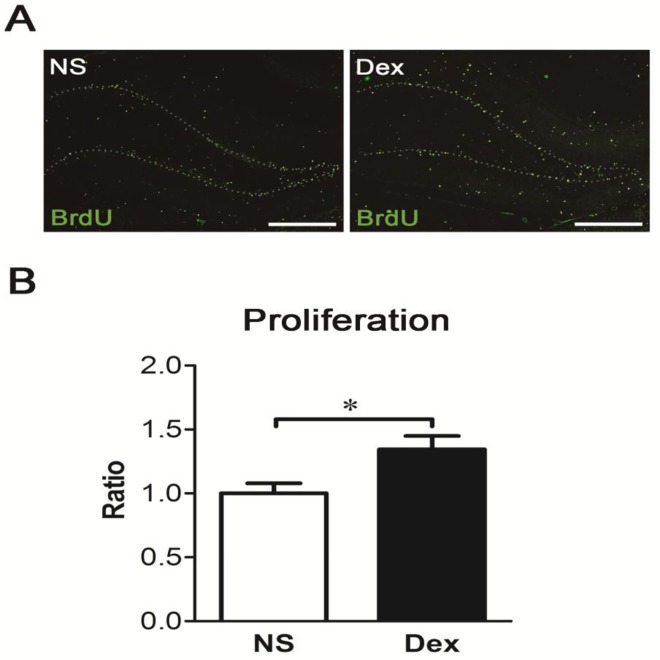
A single postnatal dose of dexamethasone (Dex) increases cell survival in the dentate gyrus. The rat pups were treated with NS or Dex at P7 followed by a single injection of BrdU (50 mg/kg, ip.) and sacrificed at P56. Coronal sections were obtained and BrdU and identified with an anti-BrdU antibody. (A) Representative images show the dentate gyrus, and BrdU-positive nuclei are characterized as cell survival (green color). The dotted lines show the shape of dentate gyrus. Bar = 500 μm. (B) BrdU-positive nuclei were counted and the number of BrdU-positive nuclei was significantly higher in the Dex group than in the NS group (Mann-Whitney test, *p < 0.05). Results are representative of the mean ± SEM (*n =* 5 for each group).

**Fig 8:**
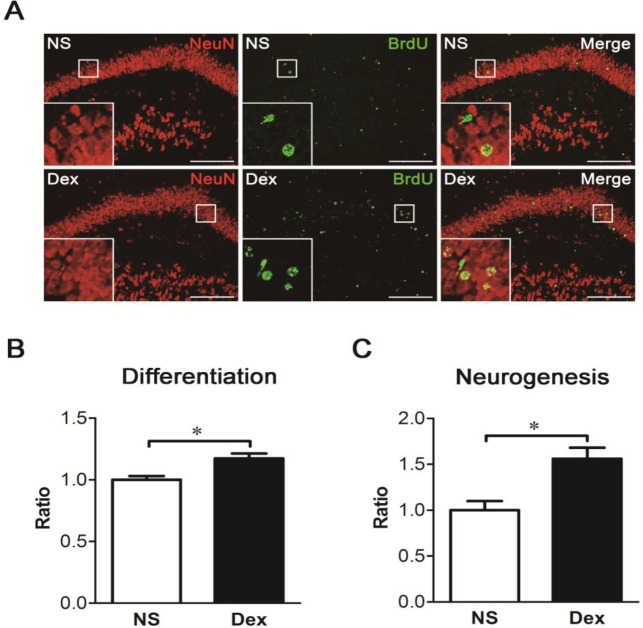
A single postnatal dose of dexamethasone (Dex) increases neurogenesis in the dentate gyrus. The rat pups were treated with NS or Dex at P7, followed by a single injection of BrdU (50 mg/kg, ip.) and sacrificed at P56. (A) To identify the neurogenesis in the dentate gyrus, double immunofluorescence staining was performed to detect NeuN (red color) and BrdU (green color). The dotted lines show the shape of dentate gyrus. Bar = 200 μm. (B) Quantification of neuronal differentiation of newly born cells. The percentage of BrdU-positive cells co-expressing NeuN was calculated, and the percentage of neuronal differentiation was significantly higher in the Dex group than in the NS group (Mann-Whitney test, *p < 0.05). Results are representative of the mean ± SEM (*n =* 5 for each group). (C) Quantification of co-localized cells in the dentate gyrus. Net neurogenesis was significantly higher in the Dex group than in the NS group (Mann-Whitney test, *p < 0.05). Results are representative of the mean ± SEM (*n =* 5 for each group).

To explore the fate of newly born cells, the rat pups were treated with NS or Dex followed by a single injection of BrdU (50 mg/kg, i.p.) at P7, and sacrificed at P56. Coronal sections were obtained, and BrdU was identified with an anti-BrdU antibody (Fig 7A). Cell survival in the dentate gyrus was significantly higher in the Dex group than in the NS group (Fig 7B, *p < 0.05). To further investigate whether these cells were neurons, the sections were analyzed with anti-NeuN and anti-BrdU (Fig 8A). Both neuronal differentiation and net neurogenesis were significantly greater in the Dex group than in the NS group (Fig 8B and 8C, *p < 0.05). This data indicates that a single postnatal dose of Dex increases survival and neuronal differentiation of newly born cells, and consequently increases net neurogenesis in the dentate gyrus at P56.

## Discussion

Dexamethasone (Dex) has been used as a therapeutic agent in preterm infants to prevent and reduce the severity of chronic lung disease (CLD) for the past three decades. However, early postnatal Dex therapy has been shown to result in adverse effects on neurodevelopment, particularly in infants treated with Dex within the first week of life [1–3,5,37,38]. It is necessary to re-evaluate the therapeutic strategies of Dex. However, the safe time points and doses of Dex for preterm patients are still undetermined.

We previously demonstrated that administering a single dose of Dex to P1 rat pups increased apoptosis in the dentate gyrus at P2 [12]. However in the current study, performance of behavioral tasks and morphology of granular cells at P56 were not impaired (Figs 1 and 3). In the retention of spatial learning, neonatal tapering of DEX treatment (0.5 mg/kg on P1, 0.3 mg/kg on P2, and 0.1 mg/kg on P3) did not disrupt memory retention in 8-week-old rats [14]. These results indicate that neonatal impairments caused by low or moderate doses of Dex may be only temporary. In this study, administration of Dex at P7 increases the dendritic spine density in granular cell, the surface protein expression of NMDA receptors, cells proliferation and neurogenesis in dentate gyrus, and regulates PI3K and NF-κB cascades in the hippocampus. Taken all the findings together, we speculate that precondition the hippocampus with Dex treatment at P7 may modulate its response to stress such as contextual or cued-fear test later in life. Stress-induced precondition resulted in better performance of contextual fear learning in rat [39]. In addition, stress experience created by maternal deprivation in early life may program hippocampus to enhance its learning under stressful environment later in adult [39,40]. In this study, both groups were cared with the same condition except medication. Though no functional evidence to support the results in this study, we speculate that it may reflect a vulnerable phenotype in the Dex-treated rats. Studies have shown that administering low or moderate doses of glucocorticoid facilitates memory consolidation and memory storage in rodents [41,42], and the beneficial effects may be caused by the upregulation of the serum- and glucocorticoid-inducible kinase (*sgk*) gene [43].

Dex leads to suppression of cortisol secretion by negative feedback inhibition, and suppresses the hypothalamic and anterior pituitary level secretion of ACTH [44]. During early postnatal development, neonatal pups undergo a reduction in the hypothalamic-pituitary-adrenal (HPA) response to environmental stressors [24,25]. This early period is characterized by the stress hyporesponsive period (SHRP) [28], which occurs from P4 to P14. During this period, glucocorticoid levels descend to their lowest at P9 [26]. Therefore, exogenous glucocorticoids may affect glucocorticoid-sensitive tissues during this period [27,28] and modulate its response to stress test such as contextual or cued-fear test later in life. It is likely that the treated P7 rats may produce higher glucocorticoid levels which enhance their learning ability. Although glucocorticoid receptors are found throughout the brain, there is a neural stem cell pool in the subgranular zone. Glucocorticoid receptors are enriched on the membrane of neural stem cells, whose proliferation increases after birth and reaches a maximum at P6-P8 [29]. Therefore, we reasoned that Dex produces its effects mainly in this cellular population that is undergoing proliferation at P7. As expected, the number of proliferating cells was significantly higher in Dex-treated rats than in NS-treated controls (Fig 6). Furthermore, we found that administering a single dose of Dex in the middle of the SHRP (P7) significantly enhanced the contextual freezing response, but the cued freezing response was not changed at P56. We have confirmed the effect of Dex on the function of the hippocampus by testing performance in the Morris water maze (Fig 1C and 1D). These findings are inconsistent with a number of studies, which have showed that Dex therapy in the preterm infants is associated with adverse neurodevelopmental outcomes [1–3,5,37,38]. The discrepancy may be due to different time points of treatment and the administered dose of Dex. The reference studies focus on treatment before SHRP or at early SHRP, and the present study focus on treatment at middle SHRP. Late or delayed postnatal corticosteroids for chronic lung disease in preterm infants, may reduce neonatal mortality without significantly increasing the risk of adverse long-term neurodevelopmental outcomes. However, the long-term outcome is limited in some cases [45–47]. All of the above studies suggest that the time points and doses of Dex treatment are crucial to neurological function, and need to be evaluated.

In the present study, we found the dendritic spine densities in the Dex group were significantly increased (Fig 3B). PSD-95, a protein component of postsynaptic density (PSD), could modulate spine density and is regulated by NF-κB [19,20,48]. The p50:p65 heterodimer of NF-κB shows constitutive activity in the neuron, and p65 is associated with learning and memory [21,22]. While extracellular stimuli induce synaptogenesis, the p65 subunit of NF-κB is required for upregulation of dendritic spine density [19]. The p65 subunit would be acetylated, and DNA binding affinity and transcriptional activity of acetylated p65 were enhanced [49]. Fear-conditioning tasks for example, induce elevations in histone acetyltransferase (HAT) activity and acetylated p65 [50]. There have been many studies that examined the p65 subunit of NF-κB and PSD-95 regulate spine density during development [19,20,30], but there is no effect when synaptogenesis reaches a plateau [19,31]. In this study, we found a single dose of Dex therapy at P7 increased spine density in rats at P56, and this was accompanied by an increase in the p65 subunits of NF-κB and PSD-95 (Figs 4A and 5C). This finding indicates the developmental synaptogenesis has not reached a plateau in P7 rats. However, the mechanisms would be better addressed. Further studies are warranted to investigate the role of p65.

The present study confirmed that administration of Dex at P7 increases the expression of PSD-95 and NR2A/B subunits in the hippocampus in young adults (Fig 4A, 4D and 4E). The cytoplasmic tails of NMDA receptors could interact with PSD-95, and PSD-95 stabilizes the NR2A/B subunits to the cell membrane, preventing the subunits from internalizing. Thus, responses of NMDA receptors are enhanced by PSD-95 [51,52]. A previous study showed the co-localization of the NR2A/B subunit and PDS-95 in cultured rat hippocampal neurons [53]. According to aforementioned data, hippocampal binding of PSD-95 and NR2A/B may be enhanced in the Dex group.

We showed that administration of Dex at P7 increases the expression of PI3K subunits in the hippocampus in young adulthood. Selective stimulation of NMDA receptors promotes the trafficking of PSD-95, which requires activation of BDNF and PI3K [18]. Phosphorylation of the NR2B subunit has been observed after long-term potential stimulation [54–56], and our results suggest that increased expression of PI3K promotes the phosphorylation of NR2B subunits.

In summary, we found that administration of Dex at P7 increases contextual fear and spatial learning later in adult life. The behavioral improvements were accompanied by enhancements in cell survival, neuronal differentiation, and synaptogenesis in the dentate gyrus. The beneficial effects are supported by increased expression of PSD-95 and NMDA receptors through the PI3K or NF-κB pathway. This finding provides evidence for the beneficial effect of a single dose of Dex treatment at a specific stage, which may open a new avenue for exploring the impact of using steroids in newly born infants.
